# Equine Asthma: Current Understanding and Future Directions

**DOI:** 10.3389/fvets.2020.00450

**Published:** 2020-07-30

**Authors:** Laurent Couetil, Jacqueline M. Cardwell, Renaud Leguillette, Melissa Mazan, Eric Richard, Dorothee Bienzle, Michela Bullone, Vinzenz Gerber, Kathleen Ivester, Jean-Pierre Lavoie, James Martin, Gabriel Moran, Artur Niedźwiedź, Nicola Pusterla, Cyprianna Swiderski

**Affiliations:** ^1^College of Veterinary Medicine, Purdue University, West Lafayette, IN, United States; ^2^Department of Pathobiology and Population Sciences, Royal Veterinary College, London, United Kingdom; ^3^College of Veterinary Medicine, University of Calgary, Calgary, AB, Canada; ^4^Cummings School of Veterinary Medicine, Tufts University, Grafton, MA, United States; ^5^LABÉO (Frank Duncombe), Normandie Université, UniCaen, Caen, France; ^6^Department of Pathobiology, University of Guelph, Guelph, ON, Canada; ^7^Department of Veterinary Sciences, University of Turin, Grugliasco, Italy; ^8^Vetsuisse Faculty, Institut Suisse de Médecine Équine (ISME), University of Bern and Agroscope, Bern, Switzerland; ^9^Faculty of Veterinary Medicine, University of Montreal, Montreal, QC, Canada; ^10^Meakins Christie Laboratories, McGill University Health Center Research Institute, Montreal, QC, Canada; ^11^Department of Pharmacology, Faculty of Veterinary Sciences, Universidad Austral de Chile, Valdivia, Chile; ^12^Department of Internal Diseases With Clinic for Horses, Dogs and Cats, Wroclaw University of Environmental and Life Sciences, Wrocław, Poland; ^13^Department of Medicine and Epidemiology, School of Veterinary Medicine, University of California, Davis, Davis, CA, United States; ^14^College of Veterinary Medicine, Mississippi State University, Starkville, MS, United States

**Keywords:** inflammatory airway disease, heaves, recurrent airway obstruction, phenotype, endotype, biomarkers

## Abstract

The 2019 Havemeyer Workshop brought together researchers and clinicians to discuss the latest information on Equine Asthma and provide future research directions. Current clinical and molecular asthma phenotypes and endotypes in humans were discussed and compared to asthma phenotypes in horses. The role of infectious and non-infectious causes of equine asthma, genetic factors and proposed disease pathophysiology were reviewed. Diagnostic limitations were evident by the limited number of tests and biomarkers available to field practitioners. The participants emphasized the need for more accessible, standardized diagnostics that would help identify specific phenotypes and endotypes in order to create more targeted treatments or management strategies. One important outcome of the workshop was the creation of the Equine Asthma Group that will facilitate communication between veterinary practice and research communities through published and easily accessible guidelines and foster research collaboration.

## Introduction

The effort to clarify the phenotype and terminology used to characterize horses with chronic inflammatory airway disease started in 2000 with a workshop in East Lansing, Michigan (1). Several workshops were subsequently held with similar goals in mind with the latest hosted in Cabourg, France in 2014 (2). In the last few years, the terminology has further evolved with the term equine asthma (EA) now being recommended to describe horses with chronic respiratory signs ranging in severity from mild to severe that were previously referred as inflammatory airway disease and recurrent airway obstruction, respectively (3). Although strong evidence supports the role of exposure to environmental dust in the pathophysiology of both mild and severe EA, the potential role of infectious agents (bacterial and viral) has not been clearly established.

The goal of the 2019 Havemeyer Workshop on Equine Asthma was to bring together researchers and clinicians from different disciplines who are actively investigating airway inflammation to discuss the latest information on this topic and provide some comparative perspective from human asthma. The workshop was designed to facilitate productive discussions that would inform potential future revisions of the 2016 American College of Veterinary Internal Medicine (ACVIM) Consensus Statement on mild-moderate EA (3) and provide future research directions.

The present report follows the format of the workshop. The manuscript is organized thematically starting with the recent advancements in the understanding of the classification and diagnosis of human and equine asthma. The second part is centered on the etiology and pathophysiology of EA. The third and final section of the manuscript summarizes the extensive discussions conducted during the workshop with the goal of prioritizing future directions of EA research.

## Clinical and Molecular Phenotypes of Human Asthma—James Martin

Clinical asthma phenotypes have been recognized for many decades but were collapsed into a unified hypothesis of asthma as an allergic disease in 1989 when age adjusted levels of immunoglobulin E were associated with asthma. It has taken more than 20 years to consider the heterogeneity of asthma again with an emerging emphasis on endotypes, an intrinsically more interesting approach to understanding asthma pathobiology (4). The term “endotype” is used to describe a subtype of disease defined by a molecular mechanism, genetic variation or by treatment response (5, 6). Cluster analyses of asthma cohorts have revealed groups with different ages of onset, lung function, concordance or lack thereof between measures of airway inflammation by sputum analysis and symptoms. A recent review of asthma by a panel of experts has focused on the need to recognize asthma in its diverse forms and to identify treatable traits. This extensive review has highlighted areas for future attention (7).

The application of the analysis of gene expression to airway epithelial cells and sputum cells from well-characterized groups of asthmatics has led to the appreciation of asthma associated with T helper 2 cytokines and non-T2 asthma (8). The former is the more allergic subset with higher IgE and peripheral and sputum eosinophilia. Non-T2 asthma has fewer of these features and is less responsive to inhaled corticosteroids. T cells that express interleukin-17 have been linked to severe neutrophilic asthma. These so-called Th17 cells have been shown in animal models to be associated with steroid-unresponsiveness. The Th1 cytokine interferon-γ likewise has been found to be expressed in the airways of severe asthmatics.

In recent years there has emerged another lymphoid cell that participates in host responses to mucosal injury. These innate lymphoid cells are lineage negative, lacking the usual lymphocyte surface markers (9). They express similar panels of cytokines to the T helper subsets and are labeled innate lymphoid cell (ILC) 1, 2, and 3. They are rapidly activated by epithelial signals such as thymic stromal lymphopoietin (TSLP), interleukins 25 and 33, molecules termed alarmins. The secretion of IL-5 and IL-13 by ILC2 may lead to a pattern of inflammation previously interpreted as Th2. Innate lymphoid cells are less steroid sensitive. Additionally, alarmins prime cells such as dendritic cells and therefore may have a role in adaptive immunity as well as innate immune responses. The synthesis of amphiregulin, an epidermal growth factor receptor ligand, by ILC2s but also Th2 cells, is postulated to promote mucosal integrity. One could anticipate that viral infection of epithelial cells or damage by irritants giving rise to inflammation mediated by ILCs. However, their roles have yet to be fully explored.

Transcriptomic analysis of sputum has revealed three patterns of inflammation and gene signatures consistent with both Th2 and ILC2 driven inflammation and oxidative stress (10). The descriptions of molecular mechanisms of inflammation may still be considered as a deeper form of phenotyping. However, the application of novel biologics to treat asthma is now implicating certain pathways in disease and therefore is providing us with true disease endotypes. Most of the progress in the identification of treatable traits has related to the T2 phenotype. Biologics targeting IgE (omalizumab), IL-5 and therefore, the eosinophil (mepolizumab, benrazilumab, rezlizumab) and the T2 cytokines (dupilumab) have all demonstrated efficacy in reducing exacerbations of asthma. Recent results of studies targeting the alarmin TSLP and therefore both T2 high and low asthma have confirmed efficacy against acute attacks of asthma. Oxidative stress in asthma has not been specifically addressed. A problematic form of asthma is that associated with airway remodeling and fixed airway obstruction. The association with mucus plugging and eosinophilic inflammation has been recently identified as a potential factor in long term impaired airway function (11).

Severe equine asthma is typically a neutrophilic form of asthma although expression of T2 cytokines has been described (12). There is also evidence that IL-17 is expressed in equine asthma and its effects on neutrophil survival are steroid-insensitive (13, 14). Although neutrophilic human asthma is less steroid-sensitive than the eosinophilic phenotype, severe equine asthma is responsive to steroid treatment despite the presence of neutrophilic inflammation. Severe equine asthma shares the structural remodeling of the airways with human asthma and a part of the remodeling change is reversible with steroid treatment as well as withdrawal from the inciting stimulus (15). Studies of airway remodeling in human asthma with treatment have not addressed key components of remodeling such as increased airway smooth muscle mass.

## Clinical Phenotypes of Equine Asthma—Jean-Pierre Lavoie

The purpose of the 2007 ACVIM Consensus Statement on mild-moderate EA was to review the current knowledge and opinions concerning this condition and to help practitioners differentiate mild-moderate EA from severe EA (16). The consensus was revised in 2016 and discussed the use of EA to describe these conditions (3). The revised consensus recognized that asthmatic horses of all severities have common clinical presentations (such as chronic cough, excess mucus, poor performance) but also a wide heterogeneity in terms of triggering factors, severity, and pathologic characteristics.

A phenotype is the observable physical properties of an organism, including measurable laboratory findings, which is the result of the expression of the genes in response to the environment (17). Identifying distinct phenotypes is of interest if they facilitate the diagnosis, the prognosis or allow the implementation of targeted therapy. While currently loosely defined, the EA phenotypes discussed in the 2016 Consensus statements were based on clinical presentation (severe vs. mild/moderate), triggering factors (barn/hay or pasture), endoscopy findings (mucus) and bronchoalveolar cytology.

From a clinical standpoint, further dividing EA as distinct “mild” and “moderate” phenotypes may promote recognition that asthma is an underdiagnosed cause of exercise intolerance in high performance horses. Horses with a cough or increased respiratory rate at rest or following exercise will commonly undergo further diagnostic procedures to confirm asthma, or “anti-asthma” treatments will be implemented. However, this is generally not the case when no clinical signs suggestive of an airway disease are present. The term “mild EA” could describe the condition affecting these horses, while “moderate EA” would be used when clinical signs of airway disease (such as cough) are present, but without the periods of labored breathing at rest seen in “severe EA” (18). The inflammatory airway cell phenotypes (neutrophils, mast cells, eosinophils) were recognized in the 2007 and 2016 consensus statements (3, 16). Future phenotypes may include the age (early or late) of appearance of clinical signs, or specific remodeling features affecting the airways, if these new features are shown to facilitate prognostication or the implementation of specific therapy.

The future development of new portable and sensitive devices for measuring the lung function of horses (forced oscillation or flow interruption techniques), or the discovery of blood biomarkers for EA would help not only to facilitate the diagnosis of mild and moderate forms of EA in clinical practice, but also to possibly identify new phenotypes for these conditions.

To date, different inflammatory pathways have been proposed as contributing to EA, which may eventually lead to novel therapies (19). The discrepancies between results of the different studies may be an indication of different endotypes in EA, although future studies on large cohorts of horses from multiple sites would be required before specific endotypes can be recognized. Multicenter tissue banking could facilitate these studies.

In summary, the 2016 ACVIM Consensus Statement recognized the currently known distinctive features of EA. Further defining “mild” and “moderate” EA based on the presence or absence of easily identified clinical signs may promote the investigation of the subclinical (mild) phenotype. The identification of novel phenotypes and endotypes may lead to “precision medicine” where treatments most likely to help equine patients would be selected. This approach is now implemented in humans and may eventually be applicable to horses if supported by scientific research.

## Summer Pasture-Associated Severe Equine Asthma: Phenotype and Triggers—Cyprianna Swiderski

Severe equine pasture asthma (EPA) is characterized by episodes of reversible airway obstruction in horses grazing pasture during the summer in hot humid climates (20). Affected horses demonstrate neutrophilic airway inflammation, airway hyper-responsiveness extending throughout the season of remission, and airway remodeling (21, 22). The author's experience is restricted to EPA as first described in horses residing in Louisiana, and diagnosed in states with subtropical climates (Mississippi, Alabama, and Florida) (20). Veterinarians in regions of adjoining states and distant states (Oregon) describe similar signs in horses grazing pastures during hot humid conditions. EPA is described in the United Kingdom where it differs in its association with hot dry weather or exposure to dust from harvest/burning of crops (23). EPA demonstrates adult onset (12 ± 6 years; range 1–29 years) without sex predilection (24). Asthma exacerbations generally begin in summer (July), persisting until temperature and humidity decrease (October/November) (25). Fewer horses experience asthma in the spring. A history of prior seasonal cough and/or exercise intolerance may be identified. Improvement within hours to days of isolation from pasture particulates in a stall environment is a key diagnostic feature of EPA in the southeastern USA (20); some severe cases necessitate isolation in a climate climate-controlled environment. In the author's experience, without adequate environmental management, disease severity is progressive and responsiveness to parenteral corticosteroids decreases.

Though specific agent(s) that elicit EPA exacerbation are not identified, the response to stall housing implicates seasonal pasture-associated particulates. Costa et al. reported increases in grass but not tree pollens were significantly associated with EPA exacerbation using a pollen station ~90 miles from affected horses (25). In this regard, intact pollen is too large to reach the respirable zone of humans in order to elicit asthma, but moist conditions that are associated with EPA exacerbations can shatter pollen and disseminate respirable particles (26). Grass pollen sensitization is classically associated with Th2 responses, IgE-mediated hypersensitivity, and eosinophilic inflammatory infiltrates. However, chronic exposure to Th2 sensitizing antigens and to complex antigen combinations that include Th2 sensitizing antigens each generate Th17 responses accompanied by neutrophilic airway inflammation that typifies EPA (27, 28).

Subtopical grasses differ substantially from grasses in temperate and continental climates (29). Pollen from subtropical grass subfamilies is important to rhinitis and human asthma in subtropical zones of Australia, Asia, India, Africa, and America. Pollination seasons for Bahia and Bermuda grass (spring through September/October) align to the season of EPA exacerbation (30, 31). The pollen season for Johnson grass is temperature dependent, flowering from May to July, with higher temperatures moving flowering later into Autumm (32).

A role for fungal triggering in EPA exacerbation is suggested by the near identical clinical picture presented by horses with EPA and barn dust-associated severe asthma, wherein a role for fungal triggering is substantiated in the latter (33). Chronic neutrophilic airway inflammation characterizing both forms of severe equine asthma also aligns to Th17-mediated neutrophilic inflammation in fungal asthma models (34). Of the more than 100 species of fungi that exist in biotropic relationships with Bermuda, Bahia, and Johnson grasses, *Curvularia, Helminthosporium, Alternaria, Puccinia, Epicoccum*, and *Fusarium* are implicated in eliciting human asthma (35). Costa et al. identified fungal spores of the genus Nigrospora, and Curvularia, as well as basidiospores, as temporally associated with exacerbations of pasture asthma (25). These findings are in agreement with reported correlations between EPA exacerbation and high dew point temperature (25). Specifically, *Nigrospora* conidia and basidiospore release increase with increasing relative humidity, resulting in a peak in spore counts during the early morning and aligning to the association of EPA exacerbations with increased dew point temperature (25, 36). In contrast, conidia of *Cladosporium, Alternaria, Epicoccum*, and *Dreschlera* spp. are released during warm, dry, windy conditions, while precipitation is required for release of many ascospores. In this way, humidity influences fungi of relevance to asthma in different locales which could influence associations of pasture asthma in the UK with hot dry conditions, rather than hot humid conditions precipitating pasture asthma in the southeastern US.

As a chronic and progressive disease of undetermined etiology, EPA is most effectively managed by segregation from inciting grass pastures during warm seasons. The necessity to segregate horses from pasture, particularly at a time when they are typically extensively ridden and grazed, presents a conundrum that is ultimately detrimental for most affected horses. Accordingly, there is a critical need to identify the agents that trigger EPA in order to improve disease management.

## Recommended Minimum Database for Diagnosis of Equine Asthma by Equine Practitioners in the Field vs. Criteria Used for Research—Melissa Mazan

Both veterinary practitioners and researchers muse about the diagnostic armamentarium available to physicians—if only we had the chest CT, the advanced lung function testing, the biomarkers—then we would be able to have a better diagnosis. A quick search of the literature, however, shows us that our counterparts face many of the same diagnostic dilemmas that we do, albeit often with higher bills! While pulmonologists have drawn up multiple guidelines to help in the diagnosis of asthma in humans with its multiple phenotypes and endotypes, physician-diagnosed asthma criteria often fail to be consistent with the official guidelines rendering the results of large epidemiologic studies or clinical trials fraught with the perils of resting findings on nebulous datasets. Various forms of spirometry or simple pulmonary function testing are readily available in human medicine, but few non-pulmonologists avail themselves of objective data, and instead rest on reported symptoms such as difficulty breathing on exertion, cough or positive response to bronchodilation (37). Indeed, the GINA toolbox identifies “lack of access to spirometry/bronchoprovocation tests” as a barrier to implementation of GINA guidelines in human asthmatics (38). Moreover, the heterogeneity in published algorithms for diagnosis of asthma—more than 66 in the literature at last count—make even an algorithm-based diagnosis unsure (39). Thus, the conclusion that symptom-based diagnosis is associated with a significant risk of over-diagnosis has been reached for asthma in humans (40). The current push in human medicine to refine both the phenotypes and endotypes for multiple different subtypes of asthmas aims to elucidate the underlying causes and thus treatments that may be very different. We are still searching for the criteria that will help us with this in equine medicine. If there are indeed mechanistically different groups of horses within the categories of mild, moderate, and severe EA that are associated with genetic differences or cellular or molecular biomarkers, then perhaps we will gain better understanding of treatment successes and failures and will be able more logically to choose clinical therapies and predict responses.

The difficult case for the clinician and the researcher alike is not the horse with severe EA—because the history and clinical exam alone can often suffice to diagnose, and there is a visible relief in respiratory embarrassment with administration of bronchodilator (although it can take some time in horses with diaphragmatic exhaustion) (41). The difficult horse is the one with moderate/severe asthma in remission and the horse with mild-moderate EA. As was recently pointed out, the biggest difference that we note in the clinical diagnosis of horses with mild-moderate EA vs. severe EA is the presence of an increased respiratory effort at rest, which is due to the underlying pathophysiology of bronchoconstriction, increased mucus, and bronchiolar inflammation (42). The need, then, is to detect the mildly or subclinically affected horse.

As veterinarians, we have at hand history, clinical signs, lung function testing, radiographs, endoscopy, analysis of airway secretions, blood biomarkers and clinical pathology which can be used in a minimum database in order to classify horses into clinically useful categories that have a pathophysiologic basis that can simultaneously allow us to diagnose, treat, and translate clinical cases into field research.

### History

A tentative diagnosis of EA in its most severe form can often be made on history alone, with the key component being the recognition of episodes of reversible respiratory embarrassment precipitated by exposure to specific triggers—namely, moldy hay in the northeast of the United States, and pasture allergens and particulates in the south. History in subclinical or mild cases is seldom of such definitive use; this does not mean that it is unimportant. Such questions as parentage (43), type of feed and how it is fed (44), and heat and pollen counts at the time of diagnosis (45) may be important risk factors for equine asthma. While it has been proposed that coughing and poor performance may serve to define a phenotype of moderate vs. mild EA (46), these signs are not sufficiently sensitive (47) and would misclassify a subset of horses—they alert the clinician that moderate EA is likely, but the absence of these signs does not rule out disease. The connections between EA and viral or bacterial disease are not linear, but it is becoming increasingly clear that the connection exists (48, 49), thus a thorough history should include probing for past infectious respiratory disease. One of the best described questionnaire analysis tools for classification of horses based on history is the HOARSI index (50), developed as a means of distinguishing among normal, mild-moderate EA or severe EA phenotypes. However, clinical signs and indices are insufficiently sensitive to distinguish horses with mild-moderate EA from normal horses or horses with severe EA in remission (51).

*Proposed minimum database for both practitioners in the field and for research: A common history tool should be developed that addresses the main concerns of parentage if known, current and lifetime exposures to particulates and allergens including feeds and feeding practices, barn environment, vaccinations, travel history, and recent illnesses*.

### Clinical Scoring/Clinical Presentation

Multiple scoring systems have been shown to be useful for distinguishing healthy horses from horses with severe EA in exacerbation, but, similar to questionnaire indices, these scoring systems do not help in the more difficult problem of distinguishing horses with mild EA from healthy or severe EA in remission (52). Indeed, 19 years ago, Robinson et al. found that even in horses with historical severe EA, clinical score failed to reflect low-grade airway obstruction, and suggested that without easily used, field-accessible testing equipment, lower airway disease would go underdiagnosed (53). Recently, the adapted 23-point scoring system has been shown to be the most useful in discriminating mild from severe cases, but it is unlikely to distinguish normal from subclinical disease (54), and the IDEASS scoring system has recently been described as a useful scoring system for moderate-to-severe equine asthma (55). Thus, while clinical scoring is essential to a good examination and careful research, and can potentially be useful in measuring response to treatment in the individual, it is insufficient in making the phenotypic distinction between mildly affected horses and healthy horses.

*Proposed minimum database for both practitioners in the field and for research: The 23-point modified clinical score appears to best stratify horses with obstruction ranging from mild to severe. An application suitable for smart phone use would enhance the adoption of a common scoring tool*.

### Lung Function Testing

In human asthma, the gold standard is the detection of variability in pulmonary function using spirometry or other methods of lung function testing (38). Unfortunately, lung function testing remains available only to a few specialized centers, as more recently developed portable lung function testing modalities are no longer on the market (41). Initial reports from the author's laboratory of a simple field test of respiratory resistance using the interrupter technique hold promise for increased use of lung function testing in the future. While the classic esophageal balloon/pneumotachometer method is effective in demonstrating increased maximal pleural pressure and allows for calculation of pulmonary resistance and elastance as well as dynamic compliance in severe EA, it is not sufficient for demonstrating abnormal function in mildly affected horses in which baseline lung function is rarely abnormal and histamine or other bronchoprovocation or bronchodilation must be used in order in order to detect low-grade obstruction (56). Unfortunately, in some studies, even histamine bronchoprovocation has not been sufficient to distinguish between normal horses and horses with mild asthma (52), and a lack of concordance between histamine bronchoprovocation and bronchoalveolar lavage (BAL) cytology has been noted in several studies (57, 58). While lung function testing and histamine bronchoprovocation have shown moderate to strong correlations with BAL cytology in some studies (56, 59, 60), others have not (57, 58). Methods of performing histamine bronchoprovocation are equally important: studies in human asthmatics have shown that it is the total dose of histamine that is most important rather than the duration of exposure. A more precise method of dosing may be important to establish. In human athletes, indirect stimuli, such as cold air, hypertonic solutions such as mannitol, exercise, and AMP are all considered more accurate and useful in predicting asthma than are direct stimuli such as methacholine or histamine; this is an area that requires exploration in equine pulmonology. While hay challenge is useful for research in severe EA, it is inappropriate in a clinical case, especially in a horse that is expected to do athletic work (3). In moderate to severe EA, variability in airflow should be demonstrated not through bronchoprovocation but through bronchodilation using either systemic (Buscopan™) or inhaled (albuterol, ipratropium bromide) drugs to assess reversibility; it is possible that a 24 h period of bronchodilation is necessary for maximum effect in horses with diaphragmatic fatigue (61).

*Proposed minimum database for both practitioners in the field and for research: In research, lung function should be assessed and airflow variability/changes in airway caliber should be assessed with either bronchoprovocation or bronchodilation. More research is necessary to determine if field assessment of lung function after bronchoprovocation or bronchodilation is sufficient to determine change with the 23-point scoring system. It is essential that a robust, easily used system for testing lung function in the field be developed*.

### Airway Secretions—Bronchoalveolar Lavage

Unlike in human pulmonology, examination of airway secretions is a primary method of diagnosis in EA, be it mild, moderate or severe. Although a standard volume of between 250 and 500 ml of saline using a 2 m long endoscope or 3 m BAL tube is recommended (3), this practice is not always followed, and cytology should be assessed keeping in mind that the amount of fluid infused will affect the cell percentages. The relationship between BAL cytology and performance is still not clear. Certainly, poor performance has been associated with what have been determined to be abnormal cell types or percentages (3). There has been much discussion as to what is normal on BAL cytology; it likely depends on a combination of technique, environment and population. Even the “stringent” definition proposed by Couëtil et al. (3) of <5% neutrophils, 2% mast cells, 1% eosinophils, would be considered elevated in some high-performance populations (62, 63). Although an earlier study found no evidence of a clear phenotype in mast cell *vs*. neutrophilic inflammation with respect to pulmonary gas exchange during exercise (64), recently, an increase in BAL mast cells or neutrophils was shown to negatively affect performance (44). The way that cells are counted in BAL cytology is also important, especially for rare cells. In our laboratory we count a minimum of 500 cells at 400x for common cells such as macrophages and lymphocytes or neutrophils in mild EA, whereas for rare cells such as mast cells we count 1,000 cells. Other techniques, such as using a 5-field differential for mast cells, are only useful if the cell density is high (65).

The conundrum of whether to assess airway fluid from both lungs rather than blind sampling, or to pool samples, has also occupied attention from researchers. One group found that, depending on whether the “loose” or “stringent” categorization was used, 8–37% of horses would have been categorized as control *vs*. mild-moderate EA if only one lung were used (66). As it is the rare practitioner who has a bronchoscope in the field, it is unlikely that even pooled samples (63), which may be a better representation of overall lung inflammation, will be taken other than in referral centers or practices. The problem is most important for rare cells. More attention will need to be paid in future to morphology and perhaps typing of cells. The existence of neutrophil extracellular traps (NETosis) in horses with severe EA presents an additional method to determine response to treatment (67), and recently the presence of degenerate neutrophils has been shown to raise suspicion for bacterial infection (68). The question of macrophage morphology as an indicator of inflammation is also an area that will profit from further investigation (69). Recently, as well, the paucigranulocytic phenotype has been described in which horses with clear signs of severe EA have low neutrophil percentages in the BAL (46). This is thought to be due to mucus plugging of small airways that essentially sequesters neutrophils. Although a recent publication showed a rather shocking 81% of high-performing European horses with mild-moderate EA had fungal elements in the BAL (62), this remains to be confirmed in other populations.

*Proposed minimum database for both practitioners in the field and for research: For the BAL, at least 250-mls of saline should be used, and there is a preference for counting at least 500 cells to adequately represent rarer cells. For research purposes where rare cells are of interest (e.g., mast cells or eosinophils), sampling of both lungs appears preferable. Better categorization of cells through morphological descriptions including apparent neutrophil extracellular traps and notations of fungal or birefringent elements should be done. Characterization of mucus on cytology may help to elucidate the paucigranulocytic phenotype. BAL in the field will usually be done blindly with a specialty tube*.

### Airway Secretions—Tracheal Wash (TW)

The debate continues to swirl around the utility of tracheal wash *vs*. bronchoalveolar lavage, with Malikides et al. (70) finding a 37% disagreement in young racehorses, while Derksen et al. (71) determining that there was no correlation between BAL and TW, and others finding no relationship between tracheal neutrophil counts and racing performance (72); thus, tracheal cytology has been considered inappropriate for diagnosis of mild EA (3). Recently, however, a comparison of TW and BAL in 145 horses, along with evidence of mucus and endoscopy, found that only 17.5% of horses would have been classified differently if they had had the other procedure, eventually concluding that there is no gold standard—except for mast cells, which are rare in the trachea, and thus, to be found, demand that a BAL be performed (73).

*Proposed minimum database for both practitioners in the field and for research: Tracheal wash may be most practical for some practitioners in the field and has the added benefit of allowing for bacterial culture. The inability to assess mast cells adequately continues to limit this modality. In research settings, both tracheal aspirate and BAL are preferable*.

### Endoscopy

Many clinical diagnoses are made on the basis of endoscopic visualization of mucus, with strong support from the finding that tracheal mucus quite nicely correlated with racing performance or lack thereof (72). The recent consensus statement considers that the demonstration through tracheobronchial endoscopy of mucus grade 2/5 in racehorses or 3/5 for sport/pleasure horses is sufficient to diagnose mild-moderate EA and in support of this recommendation, Rossi et al. (73) found that visible mucus in the trachea is indeed likely to predict inflammation. There are varying degrees of certainty about mucus in the trachea predicting inflammation (49, 62, 74, 75). Nonetheless, other studies have shown that mucus is insufficient to parse out mild *vs*. unaffected cases (76). Endoscopy has also been shown to be useful in detecting an increase in upper airway abnormalities in horses with mild-moderate EA, with Courouce-Malblanc et al. (77) raising the chicken-and-egg question of the relationship between mild-moderate EA and dorsal displacement of the soft palate, and more recently, Wysocka and Klucinski (78) found that more horses with mild-moderate EA had dynamic pharyngeal abnormalities. It may be that the answer will rest in whether any of these modalities can help to define a phenotype rather than simply further describing an already understood phenotype.

*Proposed minimum database for both practitioners in the field and for research: Upper airway endoscopy should be performed to rule out upper airway cause of obstruction as a primary cause of signs or that might confound lung function testing. Assessment of tracheal mucus should be performed*.

### Bronchial Biopsies/Brushings

Endobronchial biopsies offer an excellent method of sampling larger airways, although deeper layers cannot be accessed. The brass ring—being able to distinguish normal from remission or mild EA—remains elusive, however, as correlates were evident between histopathology and impulse oscillometry and showed a difference between horses in remission at pasture and those that remained stabled and treated with glucocorticoids, but did not show any difference between horses with severe EA in remission and controls (79).

*Proposed minimum database for both practitioners in the field and for research: At this time, brushings/biopsies are not considered part of a minimum database*.

### Radiography/Ultrasound

Imaging is considered an important ancillary diagnostic in humans, but radiographs have not been shown to be sensitive or specific in horses with EA (80). Chest CT is currently not feasible in large animals. While endobronchial ultrasound shows promise for the elucidation of airway smooth muscle thickening in severe EA, the ultimate goal of being able to detect low-grade disease in erstwhile healthy horses, or to distinguish normal from severe EA in remission remains elusive (79).

*Proposed minimum database for both practitioners in the field and for research: at this time, imaging is not considered part of the minimum database*.

## Health Effects of Equine Asthma—Laurent Couetil

Equine asthma encompasses mild to severe forms of chronic airway inflammation. Severe EA affects ~14–17% of horses in countries with Northern, cool climate (47, 81). Mild-moderate EA affects 68–77% of pleasure horses based on tracheal wash cytology (neutrophils > 20%) and up to 80% of racehorses based on BAL cytology (44, 75).

### Severe Equine Asthma

Horses affected with severe EA experience exacerbation of clinical signs when exposed to organic dust originating from hay and bedding, in particular molds present in poor quality hay. As a result, clinical signs tend to be worse during the winter when horses are housed indoors for extended periods of time (82). Some horses exhibit disease flare-ups while at pasture during summer months (EPA) (25). These horses improve clinically during winter or after being housed indoor. A small percentage of horses appear to suffer from both classic severe EA and EPA. Horses with severe asthma tend to be mature (>7 years) to old animals and a genetic predisposition has been identified in some families (83, 84).

The main clinical sign characteristic of severe EA is increased respiratory effort (“dyspnea”) that can rapidly improve following bronchodilator administration. Although the decrease in respiratory effort following bronchodilator administration can be detected within minutes of drug administration using lung function testing, clinical improvement may not be apparent to clinicians (85). Acute exacerbation is associated with increased pulmonary artery and right-heart vascular pressures as well as increased pulmonary artery diameter on ultrasound (86). Blood pressure return to baseline during clinical remission however, cardiac ultrasound abnormalities such as right ventricular wall thickness remained increased (86). Surprisingly, severe EA is rarely fatal unless complications develop such as cor pulmonale (87). Affected horses are more likely to be euthanized because owners get discouraged with the expense associated with chronic therapy and maintaining a low-dust environment (83).

Coughing and nasal discharge are non-specific signs of respiratory disease commonly reported in horse with severe EA (47). Horses with a history of both coughing and mucoid nasal discharge are at increased risk of developing severe EA (88). Thoracic auscultation may reveal increased breath sounds bilaterally, extended area of auscultation, and abnormal breath sounds (i.e., crackles, wheezes). However, the thick chest wall of horses makes auscultation an insensitive indicator of pulmonary disease, with abnormal findings obtained in <50% of horses with severe EA (88).

Strict management changes or medical therapy will results in rapid improvement in clinical signs however, if exposure to triggering factors is not addressed improvement will be short lived or incomplete (3, 15).

### Mild/Moderate Equine Asthma

This form of mild respiratory disease is mainly subclinical with horses showing non-specific signs such as intermittent coughing and poor performance (3). However, mild asthma should not be ruled out in horses that do not cough because coughing is reported in only 38% of horses with mild asthma (89). Coughing is associated with increased BAL neutrophils (56).

Poor performance and reduced willingness to perform are associated with increased tracheal mucus scores in racehorses and show-horses, respectively (72, 90). In racehorses, poor performance has been associated with increased neutrophils and mast cells in BAL fluid (44).

There is an association between nasal discharge and increased tracheal mucus in racehorses (49). However, the association between tracheal mucus and BAL cytology has not been reported yet.

## Tissue Remodeling in Equine Asthma and Functional Consequences—Michela Bullone

The term “remodeling” defines a process resulting in a tissue that is structurally and architecturally altered compared to its healthy counterpart. In asthma, structural alterations are represented by quantitative or qualitative changes of the bronchial wall components or their surrounding tissues, whilst architectural alterations refer to the skewed relationships among such structures.

Airway remodeling has been studied only in horses affected by severe EA. An increased expression of metalloproteinases and their tissue inhibitors has been recently reported in a group of horses with mild respiratory signs and BAL cytology compatible with mild EA (91). However, the possibility that the horses studied were horses with severe EA in remission of the disease was not excluded.

Almost all airway components undergo remodeling in severe EA, both in peripheral (diameter <2 mm) and central airways. The airway smooth muscle mass as well as collagen and elastic fiber deposition are increased in the lamina propria of peripheral airways during severe EA remission compared to healthy airways (92, 93). Mucostasis, mucus cell hyperplasia, peribronchiolar metaplasia, and interstitial fibrosis are more frequently detected in horses with severe EA in remission compared to controls (94). However, histomorphometric techniques revealed no differences in the number of mucus cells per mm of lamina reticularis or in the volume of stored mucosubstance in bronchial epithelial cells (95). Central airway remodeling during disease remission is less pronounced compared to what is observed peripherally. Whether airway submucosal structures are significantly altered during severe EA remission compared to control remain to be established (79, 96, 97).

Functionally, severe EA remission is associated with a normal lung function in spite of significant structural alterations of the airways. In these conditions, the respiratory resistance correlates with the amount of collagen within the lamina propria of peripheral airways (93), indicating that, in the absence of bronchospasm, peripheral airway stiffness is the major determinant of respiratory resistance in asthmatic horses. The functional implications of peripheral remodeling become more important during disease exacerbations, when most of the changes are further accentuated and the mechanics of breathing are altered (73, 94).

There is no doubt that the major determinant of airway obstruction during severe EA exacerbations is smooth muscle contraction and that central airways play a major role (98). By definition, the force produced by a muscle is proportional to its cross-sectional area. Given the increased smooth muscle mass (and cross-sectional area) during severe EA exacerbations (79), asthmatic muscle is “stronger” and able to contract the thickened lamina propria observed in severe EA, further reducing the airway lumen. Increased mucus secretions into the airway lumen also contribute to airway occlusion (99). These same mechanisms operate in peripheral airways, where the effects on lung function are somewhat blunted by the fact that their overall contribution to pulmonary resistance is low, due to their large cumulative cross-sectional area (100). At this level, the more relevant functional effects of remodeling are the loss of lung elasticity and airway-parenchymal tethering. Adequate small airway patency is guaranteed by their intimal connection to the lung parenchyma by elastic and connective fibers. When the lung inflates during inspiration, small airways are stretched and passively dilate. Remodeling of elastic fibers and of the extracellular matrix within and around the airways and in the alveolar septa alters this mechanism, preventing the smallest airways from remaining open (101). The effect is even worse during expiration, when the lungs physiologically recoil and the airway diameter physiologically narrows. With a significantly impaired expiratory airflow, part of the air that reaches the alveoli remains trapped. This leads horses with severe EA in exacerbation to breath at increasing lung volumes [functional residual capacity (102)] in the attempt to maintain airway patency, which causes lung hyperinflation and enlarged fields of thoracic auscultation (103).

## British Racing Veterinarians' Views and Practices Relating to Mild-Moderate Equine Asthma-Tierney Kinnison and Jacqueline M. Cardwell

Anecdotal evidence to date has suggested that, although BAL sampling is widely accepted elsewhere as the diagnostic tool of choice for cytological assessment of equine lower airways, tracheal endoscopy and tracheal wash-based diagnostics have remained the mainstay of routine clinical lower airway investigations in British Thoroughbred racehorses in training. Given the emphasis on BAL in research, this would present a considerable challenge to furthering evidence-based respiratory medicine in this important equine population. In a recent study we investigated British racing veterinarians' rationales for current practices, and the challenges they face in relation to diagnosing and managing racehorse airway inflammation (104).

Qualitative data were gathered through semi-structured focus group discussions designed to capture current practices and opinions relating to the diagnosis and treatment of lower airway inflammation, as well as familiarity with and views on the most recent ACVIM consensus statement (3), in which the term “mild-moderate equine asthma” was recommended. Four British veterinary practices, two primarily serving the flat racing community and two primarily serving the National Hunt (jump racing) community, in different geographical regions of England, were purposively selected to participate. Focus group discussions were conducted at the practice premises, moderated by one of the authors (TK), an experienced qualitative researcher who is not a veterinarian. Discussions were audio-recorded and transcribed verbatim, and transcripts were analyzed using an inductive, thematic analysis.

In total, 25 participants contributed to the focus group discussions (number per group ranged from 3 to11). All were veterinarians (experience ranging from recent graduate to senior partner), with the exception of one laboratory team member and one veterinary student, and five were women. Discussions lasted between 46 and 74 min.

Three key themes were developed through analysis of focus group data: (i) An over-arching theme of *serving the racing industry* within which two further themes (ii) disregarding of *the consensus* and (iii) *the pragmatic clinician* were nested.

- *Serving the racing industry*: This was a key driver of clinical approaches to racehorse respiratory health, which were strongly trainer-influenced in particular. The trainer selects horses for endoscopic respiratory assessment, often because of training and racing schedules rather than any clinical signs, and the approach to investigation and treatment is strongly influenced by trainer expectations. This varies with trainer personality, experience and training methods, as well as stage of the racing season, signalment of the affected animal and general health on the yard, and is in turn driven by commercial pressures of the racing industry.
- Disregard of *the consensus*: The unanimous view across all four groups was that the condition defined as mild-moderate EA by current concensus (3) is largely not seen in British racehorses which, in the participants' considerable collective experience, are affected predominantly with excess endoscopically-visible tracheal mucus largely attributed to bacterial infections. It was also considered unfeasible to fulfill two key aspects of the consensus case definition: waiting for chronicity of clinical signs (>3 weeks duration), and performing BAL sampling. Neither of these would be acceptable to trainers, according to participants, and participants themselves were not convinced of the extra value of BAL sampling. The consensus statement was therefore seen as having been developed for outsiders, by outsiders without sufficient understanding of culture and practices on British racing yards.
- *The pragmatic clinician*: Participants shared a strong professional identity as pragmatic clinicians often required to base clinical decision-making on direct personal or collective experience, rather than on research-based or laboratory evidence. Cytological examinations of tracheal wash samples were defended as valuable when interpreted sequentially and combined with knowledge of the history and idiosyncracies of the individual horse and yard. Although this approach was generally viewed positively as flexible and individualized, participants did also express some frustration with the sometimes unsatisfactory jigsaw of diagnostic information available to them, particularly in relation to discrepancies between clinical and laboratory findings.

Our work has highlighted a lack of alignment between clinical practice on British racing yards and international consensus on diagnosing lower airway inflammation, which constitutes a barrier to furthering development of a contextually-relevant evidence-base for this population. Equine clinicians elsewhere may find themselves in disagreement with some of the opinions expressed, or practices described, by our study participants. However, these investigations were designed to understand the experiences and rationales of clinicians in the specific context of British racing practice. The strength and consistency of views expressed support the anecdotal evidence that, in this context, tracheal endoscopy and wash sampling are widely regarded as the best available means of providing the non-invasive monitoring of respiratory health expected by trainers and used to inform training- and racing-related decisions. It would be interesting to determine whether similar approaches are being taken elsewhere, particularly in populations of yearling and 2 year old Thoroughbred racehorses in training. Given the considerable resistance to BAL sampling in British racing, development of new tracheal-based or other minimally-invasive diagnostics, including appropriate biomarkers and suitably sensitive, portable lung function tests, would be valuable. Furthermore, our participants' views that mild-moderate EA as defined by current consensus is largely not seen in British racehorses suggest that research furthering our understanding of the etiology and pathogenesis of airway inflammation in this equine population is still required.

## The Microbiome in Equine Asthma—Renaud Leguillette

The respiratory system is an interface between the outer environment and the inner body. Lower airways have historically been seen as a sterile milieu, thanks to the anatomical configuration, local surface immunity and mucus production and clearance systems (105). However, with the development of high sensitivity and high throughput technologies, the microbiota of the respiratory system has been described in healthy subjects in many species, including horses (106, 107). Further investigation of the relationship between infectious agents, lower respiratory tract microbiota and the development of mild EA is warranted. We and others have reported descriptive results about the microbiota of horses with mild EA (107, 108), but the causality between bacterial flora and the disease is far from being understood.

Studies on the microbiome use DNA extraction followed by high throughput amplification and sequencing of the 16S amplicon (109). The sequences are then filtered and aligned against a taxonomy database to identify and organize operational taxonomic units (OTUs). Descriptive analysis of the phyla, OTUs and bacterial species are then performed, followed by statistical analysis at the community level (within and between samples; alpha and beta diversity, respectively) and at the individual level (OTU diversity analysis). Statistical analysis can be used to compare between groups: healthy horses vs. those with mild asthma, upper vs. lower respiratory tract (109).

The lower airways have a decreased richness (alpha diversity, corresponding to the number and proportion of each bacterial species) when compared to the upper airways in healthy horses (107). However, a very large majority of the same OTUs are present in both the upper and the lower airways, showing an overlap and some continuity in the bacterial population between the two anatomical environments in healthy horses. Furthermore, treatment with corticosteroids did not affect the composition of the bacterial flora in the upper airways (107). The role of the upper airways microbiota in mild EA is unknown, but two studies did not find any difference in beta diversity of the upper airways between healthy horses and those with mild EA (107, 108).

The relationship between bacteria and the lower respiratory tract of the equine host seems to be dynamic. As an example, a change in the environmental respirable particulates has an effect on the lower respiratory tract flora in horses. Furthermore, treatment with systemic or nebulized dexamethasone induces some changes in the microbiota of the lower respiratory tract in both healthy and mild asthma horses (107). Systemic dexamethasone administration decreased the evenness of the flora and increased the abundance of 9 OTUs. There is an agreement between studies that the lower airways microbiota between healthy and mild EA horses are clearly different (107, 108). Interestingly, Streptococcus is one of the 6 OTUs which differed with disease status, and was the OTU with the greatest increase in relative abundance in mild EA.

The effect of the environment on the composition of the lower airways' microbiota is also a common finding between studies (107, 108). However, a study found that treatment with corticosteroids had more effect on the composition of the bacterial flora than changes in the environment (107).

The microbiome studies are recent in equine medicine and are limited to being descriptive. The challenge for the scientific community will be to answer the causality dilemma of the chicken or the egg regarding the role of the airway microbiota in mild EA.

## Role of Viruses in Equine Asthma—Nicola Pusterla

### Human Asthma

Asthma development in humans is most probably caused by the interaction of multiple factors, including genetics, allergen exposure, microbiome and invading pathogens. Human rhinovirus, human respiratory syncytial virus, human metapneumovirus, human parainfluenza virus, human enterovirus and human coronavirus are strongly associated with asthma exacerbations (110). The association between human rhinovirus-induced wheezing and the development of childhood asthma/wheezing has been confirmed in a recent meta-analysis (111). The risk for asthma by age 6 years has been shown to increase (odds ratio 9.8) if children have been wheezing with rhinovirus during the first 3 years of life (112). Further, many prospective long-term follow-up studies have shown that human respiratory syncytial virus-induced bronchiolitis is associated with later development of asthma (113). However, the pathogenic role of respiratory viruses as triggers for the development and/or exacerbation in asthmatic human patients has not been fully characterized. Changes in the immune response to viral infections in genetically predisposed individuals are very likely to be the main factor involved in the association between viral infection and asthma (114).

### Equine Asthma

The pathogenesis of EA remains incompletely defined. However, similar to human asthma, a multifactorial process is suspected. Conditions associated with exercise, feeding and housing practices, location, seasonality, infection of the upper and lower airways and genetic influences have been linked to EA (7, 8, 115, 116). A variety of viral [equine influenza virus (EIV), equine herpesviruses (EHV) equine rhinitis viruses (ERVs)] and bacterial (*Streptococcus equi* subspecies *zooepidemicus, Actinobacillus* spp., *Pasteurella* spp.) etiological agents have been linked to mild to moderate EA (49, 117). It remains to be determined if these agents are triggers for the development of EA or are secondary colonizers of already compromised airways.

#### Evidence for Viruses in Equine Asthma

Viral respiratory infections are one of the most common health problems in horses throughout the world (Table 1). These infections are often self-limiting and a full recovery can be expected in most horses. Young performance horses, such as racing horses, have an increased risk of respiratory viral infections. This relates to age susceptibility, commingling, stress and suboptimal biosecurity protocols (119, 122, 123).

**Table 1:** Association of respiratory viruses with mild to moderate equine asthma based on antigen and/or antibody detection.

**Virus**	**Year**	**Country**	**Population**	**Sample type**	**Outcome**	**References**
EAV	2015	Sweden	Standardbred trotters	Nasal secretions	No detection by qPCR	(118)
EIV	2015	Sweden	Standardbred trotters	Nasal secretions	No detection by qPCR	(118)
	2015	USA	Adult horses	BAL fluid	No detection by qPCR	(119)
	2016	France	Standardbred trotters	Nasal secretions TW	No detection by qPCR	(120)
ERAV	2015	Sweden	Standardbred trotters	Nasal secretions	No association with poor performance	(118)
	2015	USA	Adult horses	BAL fluid	High seroprevalence titers	(119)
	2016	France	Standardbred trotters	Nasal secretions	No detection by qPCR	(120)
				TW	No association with equine asthma	
ERBV	2015	Sweden	Standardbred trotters	Nasal secretions	No association with poor performance	(118)
	2015	USA	Adult horses	BAL fluid	No association with equine asthma	(119)
	2016	France	Standardbred trotters	Nasal secretions	No association with equine asthma	(120)
				Tracheal wash	Detection by qPCR in horses with cough	
EHV-1/-4	2015	Sweden	Standardbred trotters	Nasal secretions	No association with poor performance	(118)
	2015	USA	Adult horses	BAL fluid	No detection by qPCR	(119)
	2016	French	Standardbred trotters	Nasal secretions TW	No association with equine asthma	(120)
EHV-2	2015	Sweden	Standardbred trotters	Nasal secretions	No association with poor performance	(121)
	2015	USA	Adult horses	Nasal secretions	Detection associated with equine asthma	(119)
	2016	France	Standardbred trotters	Nasal secretions	No association with equine asthma	(120)
				Tracheal wash	Detection by qPCR in horses with cough excessive tracheal mucus	
EHV-5	2015	Sweden	Standardbred trotters	Nasal secretions	No association with poor performance	(121)
	2015	USA	Adult horses	BAL fluid	No association with equine asthma	(119)
	2016	France	Standardbred trotters	Nasal secretions TW	No association with equine asthma	(120)
ECoV	2016	France	Standardbred trotters	Nasal secretions TW	No detection by qPCR	(120)
EAdV-1	2016	France	Standardbred trotters	Nasal secretions TW	No association with equine asthma	(120)
EAdV-2	2016	France	Standardbred trotters	Nasal secretions TW	No detection by qPCR	(120)

*BAL, bronchoalveolar lavage; EAV, equine arteritis virus; EAdV, equine adenovirus; NS, nasal secretions; PP, poor performance; STBD, Standardbred; TW, tracheal wash*.

Amongst respiratory viruses, only EIV and ERVs have an affinity to the lower respiratory tract, leading to airway hyperresponsiveness. Clinical signs associated with EIV are usually more severe than those seen with mild to moderate EA. Further, no association has been determined between mild to moderate EA and infections with EIV, EHV-1 and EHV-4 (118, 120, 121). This is in sharp contrast to the detection of ERVs (ERAV and ERBV), known to cause subclinical or mild clinical disease (118, 120, 121). In a recent study, horses with mild to moderate EA were significantly more likely to have a positive titer as well as higher log-transformed titers to ERAV when compared to control horses (120). In another study, the detection of ERBV by qPCR was significantly associated with coughing in Standardbred racehorses in training (118). Subclinical respiratory viral activity in horses with poor performance has been associated with EHV-2 and EHV-5 infection (118, 120). In a recent study, the detection of EHV-2 by qPCR in nasal secretions was significantly associated with mild to moderate EA (120). In another study, the detection of EHV-2 by qPCR was significantly associated with coughing and excessive tracheal mucus in Standardbred racing horses (118). These results are in sharp contrast to two recent studies performed on 66 Swedish Standardbred trotters, which were followed for 13 months via qPCR analysis of nasal secretions and serology (121, 124). Despite occurrence of poor performance and subclinical viral activity in the Swedish Standardbred trotters, the authors were unable to detect associations between EHV-2/-5 and clinical respiratory disease and/or poor performance. These conflicting results reflect the ongoing challenges in establishing causality between mild to moderate EA and gamma herpesviruses, known to be ubiquitous in both healthy and clinically affected horses.

In conclusion, associations between specific viruses detected via antigen or antibody detection and clinical signs of mild to moderate EA may suggest that viruses may play a role in triggering or exacerbating asthma. However, because some viruses are ubiquitous both in healthy and clinically affected horses or are often associated with subclinical disease, establishing causality is challenging and in need for further research.

## Role of Non-infectious Exposures in Equine Asthma—Katy Ivester

A growing body of research demonstrates the link between organic dust exposure and EA. Introduction of horses to high dust environments not only induces profound BAL fluid neutrophilia and airway obstruction in horses susceptible to severe asthma, but also significant neutrophilic airway inflammation in previously healthy horses (125, 126). Outside of the experimental exposure setting, higher dust exposure has also been associated with increased risk of tracheal mucus accumulation in racing Thoroughbreds (127).

Barn dust is a complex mixture, rich in potential sources of allergens as well as immunomodulators such as endotoxin and β-glucan (128, 129). In addition to individual horse factors such as age and susceptibility, this complexity may partially account for the heterogeneity of asthma phenotypes. Respirable particulates, nominally <4 μm in diameter, have been linked to eosinophilic inflammation in young Thoroughbreds entering race training (130) and neutrophilic inflammation in actively racing Thoroughbreds (44). Increasing respirable endotoxin exposures have been shown to provide an apparent protective effect against neutrophilic inflammation at low doses (44), while high doses of endotoxin augment the inflammatory response to particulates (131), suggesting a non-linear response to inhaled endotoxin in the horse. Mast cell inflammation has been found to be common in both young, untrained Thoroughbreds (130) and those that are actively racing (44), but unrelated to respirable dust or respirable endotoxin exposures. Instead, BAL mast cell proportions are related with respirable β-glucan exposures. Conversely, inhalable dust exposures have not been found to affect BAL inflammatory cell proportions. Thus, inhalable particulates, those nominally <100 μm in diameter, appear to be less relevant than respirable particulates in equine respiratory health.

Setting exposure recommendations will require better understanding of the dose-response to inhaled non-infectious agents across wider ranges of age, breed, and discipline through study designs that include both exposure and respiratory health outcome measures and utilize appropriate statistical tools to relate them. Advanced characterization of respiratory health, such as investigation of alveolar macrophage function and BAL fluid cytokine profiles, coupled with extensive exposure assessment is likely to offer valuable insight into EA pathophysiology and identify new targets for intervention. Miniaturization of optical particle counters has rendered real-time breathing zone exposure measurements on the horse both affordable and technically feasible. Finally, the equine airway is arguably most susceptible to particle penetration during athletic exertion due to large tidal volumes and extension of the head and neck, yet the exposures that horses sustain during exercise are largely unexplored. Such measures of exposure are complicated by the air speed and turbulence generated at the breathing zone during such activity and will require specialized sampling strategies.

## The Role of Neutrophils in Equine Asthma—Gabriel Moran

Neutrophils are key actors in host defense, migrating toward sites of inflammation and infection, where they act as early responder cells toward external insults (132). However, neutrophils can also mediate tissue damage in various non-infectious inflammatory processes. Airway inflammation is one of the primary characteristics of an asthma-affected horse's response to aeroallergens with neutrophilic bronchiolitis being the main lesion (133). The mechanism by which airway inflammation develops in EA is a multifaceted and dynamic process. Current knowledge suggests that the inflammatory component of this disease results from a combination of both the innate and adaptive immune responses (134). Generally, airway inflammation involves activation of pathogen-specific inflammatory cells, modulation of gene transcription factors, and release of inflammatory mediators (135). Within the airways, neutrophils likely contribute to bronchoconstriction, mucus hypersecretion, and pulmonary remodeling by release of pro-inflammatory mediators, including the cytokines interleukins 8 and 17, neutrophil elastase, reactive oxygen species, and neutrophil extracellular traps (NETs) (118–121). Oxidative stress in horses with asthma is evidenced by the increase in elastase and decrease in ascorbic acid concentrations in BALF associated with neutrophilia secondary to exposure to organic dust (136). The pathogenic role of NETs has been described for many infectious and non-infectious human diseases, including respiratory cases with a massive influx of neutrophils into the airways (137). Excessive NET release is particularly deleterious in lung diseases because NETs can expand easily in the pulmonary alveolar space and cause lung injury. Furthermore, NETs and their associated molecules can directly induce epithelial and endothelial cell death (138).

The mechanisms that regulate neutrophil functions in tissues are complex and incompletely understood and must be regulated with exquisite precision and timing. Timely apoptosis of neutrophils is central to the resolution of inflammation; dying neutrophils are known to stimulate their own efferocytosis, inducing macrophagic transition from a pro-inflammatory to an anti-inflammatory profile (139). Thus, dysregulated apoptosis and mechanisms of inflammation may play an important role in the pathogenesis of EA. The persistence of apoptosis-resistant neutrophils in the airways of horses with asthma may also impede timely neutrophil clearance and delay the resolution of airway inflammation. The discovery and development of compounds that can help regulate ROS, NET formation, cytokine release and clearance of airway neutrophils would be highly beneficial in the design of therapies for EA (133).

## Insights Into Equine Asthma Pathophysiology From Transcriptomics—Dorothee Bienzle

Asthma is a highly heterogeneous condition of the lung. Akin to the lining of the gastrointestinal tract, the lining of the airways is also in contact with external substances throughout life. Ingested substances generally pass through the gastrointestinal tract unidirectionally, and a careful balance between processing of digested food materials, nutrient absorption and limiting immunoreactivity is maintained during homeostasis, with well-known severe consequences of deviations in this balance. The airways function differently in that only gaseous substances normally pass into the distal alveoli and are exhaled in the reverse direction. Inhaled particulates also have to be expelled in reverse direction toward the nasopharynx by largely mechanical means or taken up by alveolar macrophages for disposition with minimal inflammatory evocation (140). Hence, a complex and selective epithelial barrier with differing functions characterizes both organs.

The epithelium lining the airways has unique composition, morphology and function throughout the lung, and is intimately connected to subepithelial structures such as the basement membrane, mucous glands, smooth muscle, fibroblasts, endothelium and immune cells. The epithelium forms a barrier between inhaled components and the subepithelial constituents, and also has to balance efficient transfer of gases with controlled reactivity to non-gaseous components. While the lesions of severe EA manifest predominantly with inflammation, smooth muscle hyperplasia and fibrosis of the peripheral airways and surrounding tissues, the larger airways are exposed to the same inhaled substances and also have morphological, functional and molecular changes (141).

Research initially focused on the role of club cell secretory protein (CCSP), a member of the secretoglobin family produced by non-ciliated epithelial cells concentrated within the epithelium at the transition from bronchi to bronchioles. Club cells are recognized as epithelial progenitor cells that can differentiate into ciliated and other specialized cells of the airway epithelium, participate in reduction of reactive oxygen toxicants through cytochrome enzymes, and their hydrophobic secreted protein inactivates a range of inflammatory mediators. Horses with severe asthma have fewer club cells and lower concentration of CCSP in airway fluids, which may be a function of chronic inflammation resulting in reduced regenerative capacity of the airway epithelium (142). Unique relative to other mammals, equids have two expressed CCSP genes that differ in 12 of 70 amino acids, and also in their interaction with hydrophobic molecules (143). Recombinant eCCSP increased neutrophil oxidative burst, phagocytosis and extracellular trap formation, lending support to the notion that loss of club cells has deleterious effects on lung health (144).

Whole transcriptomic changes in endobronchial epithelial biopsies from sites from 5th to 12th generation bronchi were investigated with next-generation sequencing. Each horse served as its own control to identify changes in gene expression associated with an inhaled challenge since inter-individual variability exceeded changes attributable to the challenge. A bioinformatics pipeline including quality control measures to account for duplicates, variable sequencing depth and dispersion was implemented, results were mapped to the equine genome, and predicted proteins were procured with a combination of software and manual approaches to assign appropriate Ensemble IDs for analyzing interactions. An overall conservative analytic approach yielded 111 genes differentially expressed in horses with severe asthma as a result of a challenge, with the majority up-regulated (145). Not surprisingly, many up-regulated genes pertained to inflammatory mediators and effectors and were well-known members of protein interacting networks. However, somewhat more surprisingly, genes with altered expression also concerned more broadly epithelial cell formation and maintenance, and the circadian rhythm, suggesting that multiple cell properties are affected in exacerbated EA at the transcriptomic level. Subsequent analysis of enriched gene sets in asthmatic horses further highlighted the importance of cell cycle regulation and repair pathways (146).

Transcriptomic studies of this nature yield a great deal of information, which requires subsequent confirmation regarding cell specificity, correlation with protein expression and function, and extension to a more robust number of affected and unaffected individuals. Albeit, there is strong evidence to indicate that the bronchial epithelium is profoundly altered during exacerbation of severe EA, and this insight offers new venues for investigating the role of specific proteins and for potential therapeutic targets (147, 148).

## Genetic Risk Factors of Equine Asthma—Vince Gerber

The entire spectrum of EA is influenced by interactions between the environment and genetics, but almost all research in this field has focused on the severe clinical phenotype.

While no specific genetic risk factors have been reported for mild to moderate forms of EA, genetic susceptibility to certain bacterial lower airway infections could potentially be relevant (149). Furthermore, mild but persistent respiratory signs such as occasional coughing and nasal discharge may represent early phenotypic indicators for an increased risk to later development of severe EA (88). This suggests that the genetics of milder forms of EA may be worth investigating in longitudinal studies.

Severe EA has been shown to be partly heritable in several breeds and has been the focus of genetic research involving family and epidemiological studies, whole-genome scans and investigation of candidate genes. Reports of marked familial aggregation of severe EA date back 70 years (150). Parent, age, and stable environment have significant additive effects that increase the risk for developing severe EA as defined by a history of persistent frequent coughing and/or increased breathing effort (43, 151). Offspring of affected sires have a more than 4-fold increased risk for developing severe EA (50).

Whole genome scans in high-prevalence families indicate two chromosome regions with a genome-wide significant association with severe EA (152). Importantly, the associations differ between the families: region ECA13 in one family and ECA15 in another family. Further association and gene expression studies indicate interleukin 4 receptor as a candidate gene in a subset of EA-affected horses. Molecular pathway analyses of genomic and proteomic data showed interactions between interleukin 4 receptor and SOCS5 upstream of an important molecular cascade involving nuclear factor κB (153).

So far, no causal genetic variant has been identified in interleukin 4. An allelic case-control genome-wide association study in the general Warmblood population revealed another region on chromosome 13. The best-associated marker was located in the protein-coding gene TXNDC11, which may be involved in regulating hydrogen peroxide production in the respiratory tract epithelium as well as in the expression of MUC5AC mucin (154). No genomic copy number variations were found to be associated with severe EA (155). Integrative analyses combining GWAS, differential expression (DE), and expression quantitative trait loci (eQTLs) were not able to uncover causative genetic variants that contribute to severe EA through gene expression regulation. However, results showed interesting similarities to human asthma with disease-associated genetic variants in CLEC16A that also regulate gene expression of DEXI (156). Furthermore, global gene expression studies of mRNA and miRNA levels in these high-prevalence families have shown impaired cell cycle regulation and CD4^+^ T cell differentiation into Th2/Th17 cells, respectively, in severe EA (157, 158).

At present, none of these associations are useful genetic markers in the general population. Most of the findings pertain to Warmbloods only, or even only to certain lines and families. The fact that the chromosomal regions and the mode of inheritance do not agree between families indicates genetic heterogeneity for severe EA: depending on the genetic make-up of affected horses, different genes confer the susceptibility for the disease. It appears that the genetic basis of severe EA is robust, but remarkably complex. Polygenic complexity, potentially with a larger number of genes that each may only contribute <10% to the total genetic effects, may make it difficult to discover causative variants. Nevertheless, the genetics of severe EA has revealed interesting links between severe EA, allergic skin diseases and susceptibility to intestinal parasites (159, 160).

## Are Pertinent Biomarkers of Equine Asthma Already Available to Practitioners and Researchers?—Artur Niedzwiedz

According to the National Institutes of Health, a biomarker is a characteristic that is objectively measured and evaluated as an indicator of normal biological processes, pathogenic processes or pharmacologic responses to a therapeutic intervention (161). In practice, biomarkers include tools and technologies that can help in understanding the prediction, cause, diagnosis, progression, and outcome of treatment of a disease. Although BAL cytology has been recognized as the gold standard for diagnosing respiratory diseases such as EA, currently, sensitive and specific biomarker tests useful in routine laboratory diagnostics are being sought. A simple biomarker capable of distinguishing between animals with lower airway infections and those with non-infectious airway inflammation would be helpful. Although the diagnosis of severe cases of EA is relatively easy, it is difficult to diagnose cases in remission or horses with a mild form of the disease. Ideally, molecular biomarkers should reflect a feature of relevant pathological processes. In addition, biomarker assessment should be easy, low-cost, technically accurate, repeatable and have an acceptable risk. Therefore, a measurement from easily obtainable body fluids or tissues is preferred, such as blood, urine, exhaled breath condensates, as opposed to BAL, transbronchial biopsy or lung biopsy (162).

Several biomarkers are present or altered in the airways or circulation of horses with asthma. Inflammatory markers such as acute phase proteins and cytokines have been studied as markers of systemic inflammation. However, the available literature on markers of systemic inflammation in horses with severe EA is not well-characterized and controversial (116, 163–165). Apart from reports on differential expression of cytokines during the course of severe EA, only a few acute phase proteins have been investigated. Haptoglobin is a suitable marker of both acute and chronic systemic inflammations, whereas high concentrations of serum amyloid A indicate acute inflammation. One study found no difference in the acute phase protein levels (serum amyloid A, c-reactive protein, haptoglobin) between horses with mild EA and those with other causes of exercise intolerance (166). Another study found elevated haptoglobin concentration in horses with mild EA (167).

Surfactant protein D is a large multimeric collagenous glycoprotein produced mainly by type II epithelial cells in the lungs and is also detectable in the serum. Serum surfactant protein D has been identified as a potential systemic biomarker for some pulmonary diseases in humans, such as idiopathic interstitial fibrosis and acute respiratory distress syndrome. Elevated serum levels of surfactant protein D have been detected in horses with mild EA (167, 168).

Circulating immune complexes are proteins that result from an immune response against an organism or antigens of various origin. In humans, circulating immune complexes are detectable in a variety of systemic disorders such as autoimmune diseases, allergies and infectious diseases (169). High levels of circulating immune complexes have been reported in horses with severe EA (165). Another study found circulating immune complexes useful for differentiating healthy vs. severe EA, and monitoring corticosteroids therapy (170).

The main group of enzymes responsible for collagen and other protein degradation in the extracellular matrix are matrix metalloproteinases (MMPs), while tissue inhibitors of metalloproteinases (TIMPs) lead to fibrosis formation. Collagen is the main structural component of connective tissue and its degradation is a very important process in development, morphogenesis, tissue remodeling, and repair. In horses with severe EA, MMPs, TIMPs, and their ratios are useful in the evaluation of the severity of respiratory disease and in identifying subclinical cases (91). Furthermore, MMP-2, MMP-9, TIMP-1, and TIMP-2 are significantly decreased after therapy with inhaled glucocorticoid therapy (171).

Exhaled breath condensate is a promising source of biomarkers of lung disease in humans. Exhaled breath condensate hydrogen peroxide concentration and pH were higher in horses with mild EA, vs. controls (69). Additionally, both hydrogen peroxide and pH had a positive association with BAL neutrophil percentage, while leukotriene B-4 demonstrated a positive association with BAL eosinophil percentage. Another study characterized the metabolomic profile of tracheal wash and exhaled breath condensate in healthy horses and those with severe EA (172). Higher concentrations of histamine and oxidant agents, such as glutamate, valine, leucine, and isoleucine, as well as lower levels of ascorbate, methylamine, dimethylamine and O-phosphocholine, were found in the group of severe EA, compared to healthy controls.

Many biomarkers of EA have been studied—some are already being used in clinical settings, while others require further studies. However, history, clinical evaluation, and BAL still constitute the basis for diagnosis of EA.

## How Do We Standardize Immunologic Laboratory Testing?—Eric Richard

Immune response has mainly been investigated in the airways of horses with severe EA and more recently mild-moderate EA, while still representing one of the futures direction for research stated in the 2016 ACVIM Consensus Statement (3). Such characterization has mostly been performed through relative mRNA expression of various cytokines in BAL fluid, while several publications also reported protein concentration in BAL fluid for few cytokines. Various methodologies for cytokine mRNA expressions have been published (e.g., SYBR Green or Taqman technology, design of primers and probes, relative quantitation, etc.).

Variation in methodologies may ultimately prevent objective comparisons between reports, as well as the implementation of prospective, multicenter studies. Such diversity should however not be considered as a scientific weakness, and methodological homogenization among the various research groups neither represents a prerequisite nor a final goal to be reached. However, evaluation of the methodological performances of different research laboratories might represent a relevant goal. In this manner, implementation of inter-laboratory comparisons based on international standards (e.g., ISO/IEC 17043 and ISO 13528) warrants further consideration.

Let's consider for example mRNA expression of two different cytokines by PCR in BALF samples. As a first and informal procedure, a simple “blind test” could be performed among up to four different teams. In this procedure, the “reference lab” will provide the three other labs with aliquots of the same sample(s). Each team will evaluate mRNA expression for these two cytokines based on their own procedures, and comparisons of the results obtained and agreement among the teams can be evaluated. This “blind test” might then be repeated on a regular basis, systematically alternating the “reference lab” within the group. In the end, the procedure will provide an objective evaluation of the results diversity among the teams, but clearly will not determine whether several teams are more efficient than others for these specific analyses.

A second and more structured procedure would require the specific synthesis of standards (mRNA for two different cytokines in this case), and the development/validation of relevant conditioning and conservation procedures. A similar group of four different labs would first evaluate their ability to detect and quantify predetermined amounts of analytical standards (evaluation of the detection, not of the sample extraction, etc.). This step is a necessary preliminary, in the absence of reference methods. A panel of at least 10 samples (previously calibrated with standards) would then be tested, including several identical ones (for repeatability) and submitted to the group (including a “self-shipment”) for testing and further statistical analyses (agreement, etc.). Once the methodological performance of the lab is considered acceptable for this panel, the procedure might then be repeated with another two cytokines and so on. In the end, the whole panel of standardized samples might allow the establishment of a labeling, accessible to any voluntary laboratory involved in equine asthma.

Mandatory considerations about such comparisons are that there is no trap, and this does not represent an overall examination of laboratories, but simple evaluations of procedures. All labs are expected to use their methodologies, whether or not the technologies are similar within the group. Among others, samples conditioning, conservation, shipment and their associated costs will represent major issues to be considered, and this should be more broadly associated with virtuous initiatives such as the Equine Respiratory Tissue Biobank.

## Future Research Directions in Equine Asthma: Systematic Summary of Suggestions From Final Roundtable Discussions—Jacqueline M. Cardwell, Melissa Mazan, Laurent Couetil, Renaud Leguillette, Eric Richard

Several group discussions were conducted during the 2019 Havemeyer Equine Asthma Workshop to identify future research priorities. Initial rotating small-group topic explorations (pathophysiology, risk-factors, diagnostic methods and phenotype definition) facilitated by members of the workshop organizing team, were followed by a final large group “roundtable” discussion of key directions for future EA research. The discussion was informed by data gathered directly from ~30 participants (i.e., all who attended the final roundtable), who were invited to propose up to three short- or long-term, focused or “big picture,” research topics or ideas that they considered to be key future research directions. These data were submitted anonymously, during the workshop, as free-text on paper and loosely arranged into broad categories for further open discussion.

**Figure 1:**
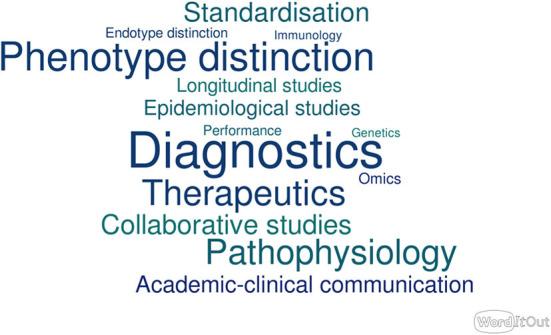
Word Cloud summary of topic areas proposed by workshop participants as key future directions for equine asthma research.

Following the workshop, in order to present an accessible, systematic and non-selective summary of the ideas proposed by participants, the free-text data were collated in Microsoft Excel for content analysis using an approach based on recommended methods for quasi-qualitative data (173, 174). The text was transcribed verbatim and coded at two levels to categorize content into (i) broad topic areas (Level 1) and (ii) specific subsets of these topics (Level 2). All instances of each Level 1 topic code were then exported into online software (WordItOut) to create a word cloud (Figure 1), in which the relative frequencies of occurrence of each topic are represented by font size.

Overall, 62 responses were received, each proposing between 1 and 3 research ideas, resulting in a total of 117 research ideas, which were organized into the 14 broad topic codes presented in the word cloud. Some research ideas encompassed more than one topic and were identified with multiple codes to reflect this. Frequencies of occurrence of each code ranged from n=28 for “diagnostics” to *n* = 1 for “genetics.”

Specific proposed areas of interest in the dominant “diagnostics” category were the development of improved, non-invasive field diagnostics through the identification of suitable biomarkers, development of portable lung function tests, improved understanding of relative values of tracheal wash in comparison with BAL cytology, or relationships between the two, and identification of gold standards for all of these diagnostic modalities.

Another key topic was phenotype distinction (21 occurrences)—in particular to clarify any distinction between mild and moderate EA, and to determine whether or not such a distinction is valuable in terms of differing pathophysiology, diagnostic indicators, therapeutics or prognosis. As with many of these proposed topics, phenotype distinction rests on the back of the category “diagnostics”—pointing out a self-identified weakness on the part of EA researchers that the goal of identifying the horse with asthma so mild that it does not present as respiratory disease *per se*, continues in many cases to elude us and underscores a collective pragmatism that there is little benefit in understanding the fine points if we cannot definitively identify the case in the first place.

Ideas relating to therapeutics (18 occurrences) included investigating the efficacy of different treatments including environmental management and any evidence for the value of antibiotics, as well as the development of optimal nebulized glucocorticoids, alternatives to corticosteroids, immunological treatments, respiratory probiotics, other novel therapeutics (e.g., MARCKS inhibitor peptide), and individualized treatments for different endotypes and phenotypes.

Suggestions relating to pathophysiology (17 occurrences) included furthering our understanding of the role of environmental pollutants, of when a physiological response becomes a pathological response and of factors influencing progression from mild to severe equine asthma.

Standardization (11 occurrences) referred in particular to the need to develop or agree on standardized diagnostic approaches, including in relation to BAL collection techniques, laboratory processing and cytological methods and threshold values, context-specific reference ranges, development of a central repository of protocols and improved quality control protocols. A central repository of standard protocols was suggested.

Academic-clinical communication (9 occurrences) was recognized as an area for general improvement. Related research suggestions included improving our understanding of the views and practices of field clinicians, as well as their perceptions of disease progression and treatment efficacy, particularly in regions outside the UK (to build on the Kinnison and Cardwell UK study) (104). This would inform the enhancement of multi-directional communication between academia, referral and first opinion clinical practice, development of guidelines and apps for field practice and overall improved dialogue and engagement.

Better use of collaborative, epidemiological and longitudinal studies was suggested for many topics and included multicenter, cross-country collaborations, more use of the existing tissue bank and the initiation of a new Equine Asthma Group.

It is recognized that the ideas for research directions generated through this roundtable discussion at the end of a 3 day workshop are subject to biases and influences relating to the interests, priorities and perceptions of workshop participants. However, by using and describing a systematic method of representing the ideas proposed, we have aimed at least to be transparent in our reporting of this. Further, longer-term, international discussion and exchange of views will be facilitated by one of the key outcomes of this workshop, which was the development of the new Equine Asthma Group. The aim of this group is to offer a platform of information for veterinary practitioners and horse owners as well as a resource for researchers to collaborate and exchange ideas on the understanding of EA. It was suggested that this group could lead some initiatives in line with the proposed areas of interest described above. There are plans for this group to develop some guidelines for the diagnosis and treatment of equine asthma, including for example the standardization of diagnostic methods, as mentioned above. Development of an Equine Asthma Group website and other communication tools are now underway as an internationally collaborative initiative.

## Conclusion

The 2019 Havemeyer Equine Asthma Workshop has paved the way for a better understanding of this many-faceted disease by bringing together researchers and clinicians to identify both the needs of the equine industry for effective treatments and at the same time focus researchers on the gaps in knowledge and understanding that will facilitate our ability to deliver on these needs. The participants made clear the requirement for more accessible, standardized diagnostics that will enable us to understand the underlying pathophysiology and identify specific phenotypes and endotypes and thus create more targeted treatments or management strategies. By creating an Equine Asthma Group, we will have a platform to unify the veterinary practice and research communities through agreed-upon research targets and through published and easily accessible guidelines, creating a point of convergence for identification of cases that will facilitate research.

## Author Contributions

LC, JC, RL, MM, and ER contributed to the editing of the final manuscript and each wrote one section of the manuscript. DB, MB, VG, KI, J-PL, JM, GM, AN, NP, and CS each wrote one section of the manuscript. All authors contributed to the article and approved the submitted version.

## Conflict of Interest

The authors declare that the research was conducted in the absence of any commercial or financial relationships that could be construed as a potential conflict of interest.
